# Challenges in Evaluating the Severity of Fibropapillomatosis: A Proposal for Objective Index and Score System for Green Sea Turtles (*Chelonia mydas*) in Brazil

**DOI:** 10.1371/journal.pone.0167632

**Published:** 2016-12-09

**Authors:** Silmara Rossi, Angélica María Sánchez-Sarmiento, Ralph Eric Thijl Vanstreels, Robson Guimarães dos Santos, Fabiola Eloisa Setim Prioste, Marco Aurélio Gattamorta, José Henrique Hildebrand Grisi-Filho, Eliana Reiko Matushima

**Affiliations:** 1 Research Group on Fibropapillomatosis in Sea Turtles, University of São Paulo, São Paulo/SP, Brazil; 2 Laboratório de Patologia Comparada de Animais Selvagens (LAPCOM), Departamento de Patologia, Faculdade de Medicina Veterinária e Zootecnia, Universidade de São Paulo, São Paulo/SP, Brazil; 3 Instituto de Ciências Biológicas e da Saúde, Universidade Federal de Alagoas, Maceió/AL, Brazil; 4 Faculdades Metropolitanas Unidas (FMU), São Paulo/SP, Brazil; 5 Laboratório de Epidemiologia e Estatística (LEB), Departamento de Medicina Veterinária Preventiva e Saúde Animal, Faculdade de Medicina Veterinária e Zootecnia, Universidade de São Paulo, São Paulo/SP, Brazil; Texas A&M University at Galveston, UNITED STATES

## Abstract

Fibropapillomatosis (FP) is a neoplastic disease that affects marine turtles worldwide, especially green sea turtles (*Chelonia mydas*). FP tumors can develop on the body surface of marine turtles and also internally in the oral cavity and viscera. Depending on their quantity, size and anatomical distribution, these tumors can interfere with hydrodynamics and the ability to feed, hence scoring systems have been proposed in an attempt to quantify the clinical manifestation of FP. In order to establish a new scoring system adapted to geographic regions, we examined 214 juvenile green sea turtles with FP caught or rescued at Brazilian feeding areas, counted their 7466 tumors and classified them in relation to their size and anatomical distribution. The patterns in quantity, size and distribution of tumors revealed interesting aspects in the clinical manifestation of FP in specimens studied in Brazil, and that FP scoring systems developed for other areas might not perform adequately when applied to sea turtles on the Southwest Atlantic Ocean. We therefore propose a novel method to evaluate the clinical manifestation of FP: fibropapillomatosis index (FPI) that provides the Southwest Atlantic fibropapillomatosis score (FPS_SWA_). In combination, these indexing and scoring systems allow for a more objective, rapid and detailed evaluation of the severity of FP in green sea turtles. While primarily designed for the clinical manifestation of FP currently witnessed in our dataset, this index and the score system can be adapted for other areas and compare the characteristics of the disease across regions. In conclusion, scoring systems to classify the severity of FP can assist our understanding on the environmental factors that modulate its development and its impacts on the individual and population health of green sea turtles.

## Introduction

Green sea turtles (*Chelonia mydas*) have a global tropical and subtropical distribution, and spend most of their lives in coastal areas foraging on seagrass and macroalgae [1,2]. Besides fisheries bycatch, habitat destruction and pollution, green sea turtles are threatened by fibropapillomatosis (FP) [3,4]. This neoplastic disease is included in the list of priority research questions based on the opinions of sea turtle researchers who work in fields related to conservation and/or turtle biology [5]. FP tumors were transmissible in experimental studies, and there is consensus that the Chelonid herpesvirus 5 (ChHV5) plays a central role in its pathogenesis [6–9]. However, the host immune response and genetic and environmental cofactors may also be involved in determining the development and clinical manifestation of the disease [10–12], and green sea turtles with many tumors can act as superspreaders in the ocean [13]. In a study in Brazilian coast with 175 fibropapillomas collected from 139 green turtles, the presence of ChHV5 was detected and quantified in 153 samples of FP tumors collected (87% were positive by qPCR) and revealed that 73% of them were positive for ChHV5 in conventional polymerase chain reaction (PCR) [14]. Phylogenetic analysis of ChHV5 variants have revealed four phylogeographical groups: eastern Pacific, western Atlantic/eastern Caribbean, mid-west Pacific and Atlantic [15], and other researchers have detected different local or regional variants, suggesting that infection occurs locally after recruitment into coastal foraging areas [9,14,16,17].

FP has a circumtropical distribution and seems most frequent in sea turtles at coastal feeding grounds, often in areas heavily impacted by human activities [9,18,19]. The disease was first described in 1938 in green sea turtles from Florida, USA [20]. In Brazil, it was first documented in 1986 at Espírito Santo state, and since then it has been reported throughout most of the country’s coastline [21].

Depending on their size, number and anatomical position, FP tumors can interfere with locomotion, food ingestion, growth, reproduction, vision, and the ability to avoid predators [22–24]. FP tumors can develop on the body surface of marine turtles, including the carapace, cornea, and plastron (where tumors develop in the sutures between the scutes), and also internally in the oral cavity, esophagus, heart, lungs, liver, spleen, kidneys, gastrointestinal system, and skeletal muscles [22,25–27]. While oropharyngeal and visceral tumors have been reported and are relatively common in green sea turtles with FP in Hawaii [26,27], they seem much less common in specimens captured in Florida [25,28] and are very rare in Brazil, where most tumors have been reported on the limbs [21,29–31].

Some studies have demonstrated that the clinical presentation of FP may be related to an individual’s growth rate, sex, parasite infestation and hematology [22,32–34], and the use of scoring systems to classify the severity of FP can assist our understanding on the environmental factors that modulate its development [4]. For this purpose, past studies have attempted to classify FP through the subjective visual inspection of specimens or of dorsal and ventral photographs [4,35,36] or through objective scoring systems based on the number of tumors and their anatomical distribution, categories of size and an estimative of the total area of tumors [22,25,27,32,33]. Among the attempts to classify FP, there is a system that uses number of tumors and tumor size category to assign a score of FP severity, which was developed using FP manifestation found in green sea turtles studied in Hawaii and has been broadly used by sea turtle conservation programs [32].

Because of a geographic variation in FP manifestation, the score system proposed by Work & Balazs (1999) [32] was not meant to be universal, being appropriate for the green sea turtles found in Hawaii. So, in order to reduce subjectivity and provide a more comprehensive framework to evaluate the severity and clinical manifestation of FP, the aim of this study was to develop a fibropapillomatosis index (FPI) and a new FP score system: the Southwest Atlantic fibropapillomatosis score (FPS_SWA_). They are herein proposed based on three aspects: (1) keeping it simple and easy-to-use; (2) to have a good correlation with total tumor area and (3) to achieve an index and a score that better assess FP manifestation in green turtles found in Brazil.

## Materials and Methods

**Fig 1:**
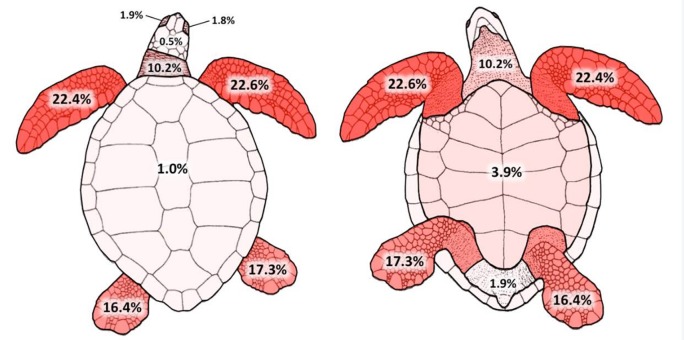
Anatomical distribution of fibropapillomatosis tumors. Percentages relate to the total number of tumors evaluated in the study (n = 7466). Drawing adapted from Pritchard & Mortimer (1999) [37].

Between 2005 and 2014, 214 juvenile green sea turtles with FP (curved carapace length ranging from 30.5 to 68.5 cm; body mass ranging from 2.6 to 35 kg) were caught or rescued along the coast of five states on the Brazilian coast: São Paulo (n = 72), Rio de Janeiro (n = 1), Espírito Santo (n = 133), Bahia (n = 4) and Ceará (n = 4) according to monitoring of Projeto TAMAR. For each examined individual, FP tumors were counted and classified into four size categories based on their diameter: A (<1 cm), B (1–4 cm), C (>4–10 cm) and D (>10 cm) [33]. Tumors of each size category were counted separately for each of eleven anatomical regions (Fig 1): left eye, right eye, head, neck, left forelimb, right forelimb, carapace, plastron, left hindlimb, right hindlimb, inguinal region/tail. Of the 214 individuals examined in the study, 124 were dead and were also examined for internal tumors: Espírito Santo (n = 113), São Paulo (n = 7) and Bahia (n = 4). For each individual, the total number of tumors (all anatomical regions combined) was calculated for each size category (N_A_, N_B_, N_C_ and N_D_; N = number of tumors in each size category A-D), as well as the total number of tumors in all size categories (N_all_). This study agreed with the Ethical Principles in Animal Research adopted by Ethic Committee in the use of animals (*Comissão de Ética no Uso de Animais*) of the *Faculdade de Medicina Veterinária e Zootecnia*, *Universidade de São Paulo* (697/2005, 1932/2010, 2116/2010 and 2555/2012) and was approved by the *Instituto Chico Mendes de Conservação da Biodiversidade (ICMBio)–Ministério do Meio Ambiente*, Brazil (SISBIO 22751, 26667, 21802 and 32636).

When we attempted to apply Work and Balazs’ classification scheme [32] to our dataset, 73% (156/214) of the studied individuals could not be classified as they fell outside the boundaries of the score categories. This is probably due to the differences in FP manifestation between turtles found in Hawaii and Brazil. An adapted version of this classification was developed, which we will refer to as the Hawaii fibropapillomatosis score (FPS_HWI_), based on three rules: (a) if an individual has no C-sized or D-sized tumors (i.e. N_C_ = 0 and N_D_ = 0) and it has five or less tumors size A and five or less tumors size B (i.e. N_A_ ≤ 5 and N_B_ ≤ 5), it is classified as ‘mild’; (b) if an individual has one or more D-category tumors and/or if it has four or more C-sized tumors (i.e. N_D_ ≥ 1 and/or N_C_ ≥ 4), it is classified as ‘severe’; (c) if an individual cannot be classified by the previous two rules, it is classified as ‘moderate’.

Additionally, we incorporated data from Rossi et al. (2009) [33] on the total tumor area (TTA) of 27 juvenile green sea turtles (a subset of the individuals examined in this study). TTA had been calculated estimating the cross-sectional area (cm^2^) of each individual tumor using the formula: π × 0.5a × 0.5b for the major [a] and minor [b] axis of an ellipse [27]. For the subset of animals for which the TTA was calculated, we performed a multiple linear regression analysis to evaluate how TTA is related to the number of tumors in each size category (NA, NB, NC, and ND). Since we sought an index that was highly correlated with TTA and simplification is a key factor to a wider use, the fitted model was then simplified by rounding its coefficients to obtain the FPI formula. After FPI was calculated for all animals, we assessed its correlation with TTA (which should be high, considering that FPI is based directly on the size classification) through linear regression. We estimated the best cut-offs for transforming this continuous variable into an ordinal one (thus creating a score for FP manifestation, the FPS_SWA_) by analyzing the scatter plot of the relationship between TTA and FPI.

In order to test which score system (FPS_SWA_ or FPS_HWI_) performed better in assessing FP manifestation in green sea turtles found in Brazil, we tested both in another subset of animals, for which an expert-based score system was implemented. A subjective fibropapillomatosis score (FPS_SBJ_) was obtained by having five researchers evaluating dorsal and ventral photographs of 52 green sea turtles (13 specimens studied in São Paulo State and 39 in Espírito Santo State), then reach a consensus to classify each sea turtle into one of three subjective categories of FP severity: ‘mild’, ‘moderate’ or ‘severe’.

The weighted Kappa statistic [38] was used to evaluate the agreement between these different scoring systems (FPS_SBJ_, FPS_HWI_, FPS_SWA_). Significance level (alpha) was 0.05 for all tests. The raw data, on which the analyses are based, are provided in S1 File. Statistical analyses were made with R software [39] and Weighted Kappa was calculated with the “irr” R package [40].

## Results

### Descriptive analysis

A total of 7466 tumors were counted, ranging between 1 and 129 tumors per individual. Most tumors were on the limbs, with 45% on the forelimbs, 34% on the hindlimbs, 10% on the neck, and 10% in the remaining areas of the body. Table 1 and Fig 1 detail the anatomical distribution of the tumors. Three of the studied specimens that were necropsied had tumors in the oral cavity and esophagus (size categories A and B, not included in statistical analyses).

**Table 1:** Anatomical distribution of fibropapillomatosis tumors on juvenile green sea turtles (*Chelonia mydas*) at select localities along the coast of Brazil. Tumors were classified in four size categories: A (<1 cm), B (1–4 cm), C (>4–10 cm) and D (>10 cm).

Anatomic regions	Mean ± S.D. tumors per turtle (by tumor size category)					Number of turtles with tumors	Number of tumors
	A	B	C	D	All		
Left eye	0.46 ± 0.72	0.19 ± 0.53	-	-	0.65 ± 0.91	88	140
Right eye	0.46 ± 0.72	0.15 ± 0.44	-	-	0.61 ± 0.83	90	131
Head	0.06 ± 0.25	0.12 ± 0.72	0.00 ± 0.07	-	0.18 ± 0.88	21	39
Neck	1.76 ± 3.04	1.67 ± 2.43	0.14 ± 0.42	-	3.57 ± 4.34	152	763
Left forelimb	3.66 ± 4.70	3.81 ± 4.17	0.32 ± 0.78	0.02 ± 0.15	7.82 ± 7.12	191	1674
Right forelimb	3.55 ± 4.54	3.91 ± 4.44	0.43 ± 0.99	0.01 ± 0.10	7.90 ± 7.28	188	1690
Carapace	0.12 ± 0.48	0.21 ± 0.82	0.02 ± 0.14	0.01 ± 0.14	0.36 ± 1.13	38	77
Plastron	0.55 ± 1.39	0.71 ± 1.29	0.08 ± 0.33	-	1.35 ± 2.23	96	288
Left hindlimb	2.30 ± 3.44	2.89 ± 3.48	0.49 ± 1.08	0.05 ± 0.28	5.73 ± 5.67	171	1226
Right hindlimb	2.87 ± 4.21	2.69 ± 3.34	0.46 ± 1.00	0.02 ± 0.18	6.04 ± 6.14	170	1292
Inguinal region/tail	0.37 ± 1.49	0.28 ± 1.06	0.02 ± 0.15	-	0.67 ± 2.06	54	144
**Total**	16.15 ± 17.26	16.65 ± 15.61	1.95 ± 3.12	0.12 ± 0.48	34.88 ± 27.13	214	7464

### Fibropapillomatosis index (FPI)

**Fig 2:**
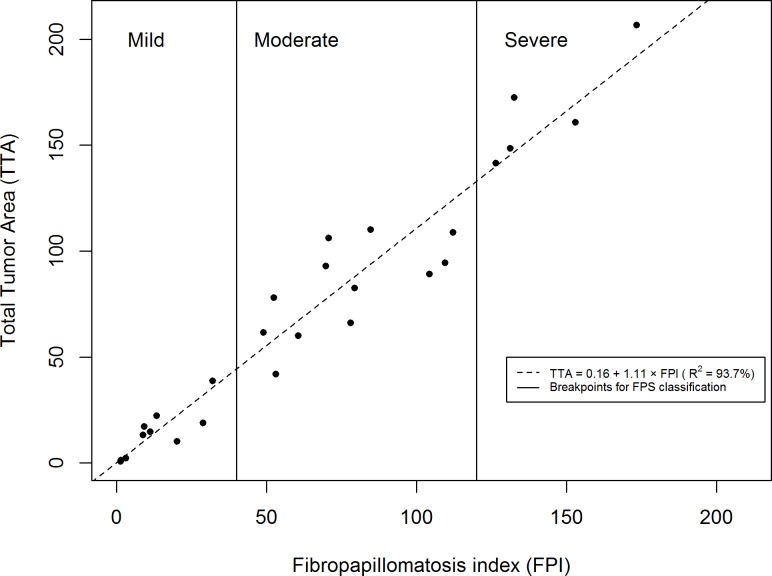
Scatter plot and fitted model of the relationship between the total tumor area (TTA) and the fibropapillomatosis index (FPI). Data obtained from 27 juvenile green sea turtles at select localities along the coast of Brazil. FPS: Fibropapillomatosis Score.

The relationship between TTA and the number of tumors in each size category (NA, NB, NC, and ND) was analyzed with multiple linear regression resulting in the following function: TTA = 0.031 + 0.056 × N_A_ + 1.172 × N_B_ + 22.161 × N_C_ + 36.198 × N_D_ (R^2^ = 93.85%). We simplified this result to obtain the following function for our proposed index: FPI = 0.1 × N_A_ + 1 × N_B_ + 20 × N_C_ + 40 × N_D_. In order to assess the correlation between FPI and TTA, we fitted a linear model with regression analysis, obtaining the following regression: TTA = 0.16 + 1.11 × FPI (R^2^ = 93.7%) (Fig 2). Considering that FPI is based directly on the size classification (taking into account only the highest dimension between length and width) and TTA is calculated with the area of each tumor, it was expected a strong correlation between them. Therefore, our proposed index was not only highly correlated with TTA, but it also made possible to have a good estimate of TTA based on FPI using the previous formula (which is much simpler than measuring each tumor individually to calculate TTA directly).

### Southwest Atlantic Fibropapillomatosis Score (FPS_SWA_)

**Fig 3:**
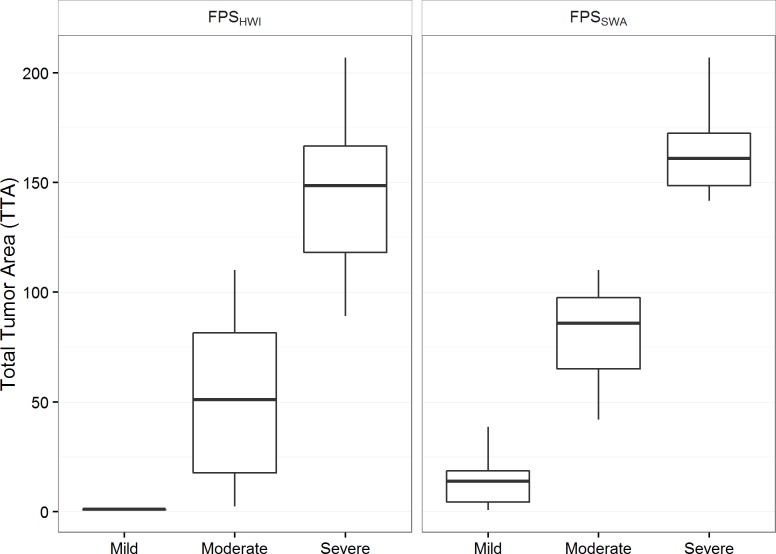
Boxplots of total tumor area (TTA) by score system: Hawaii fibropapillomatosis score (FPS_HWI_) and Southwest Atlantic fibropapillomatosis score (FPS_SWA_). Data obtained from 27 juvenile green sea turtles at select localities along the coast of Brazil.

Based on FPI, the FPS_SWA_ is proposed, wherein an individual is classified as ‘mild’ (FPI < 40), ‘moderate’ (40 ≤ FPI < 120), or ‘severe’ (FPI ≥ 120). Fig 3 shows how this score system performs in discriminating TTA from turtles on the coast of Brazil when compared to FPS_HWI_.

To further evaluate score systems, we calculated the weighted Kappa coefficient between FPS_SWA_ and FPS_HWI_ with FPS_SBJ_. This validation was made in a different subset of green sea turtles (n = 52) than the subset used previously to study TTA. FPS_SBJ_ and FPS_HWI_ had moderate agreement (κ = 50.3%), with most of disagreement being related to individuals that were classified as mild by FPS_SBJ_ but as moderate by FPS_HWI_ (17 of 28 disagreements). FPS_SBJ_ and FPS_SWA_ had substantial agreement (κ = 72.9%), with most of disagreement being related to individuals that were classified as mild by FPS_SBJ_ but as moderate by FPS_SWA_ (5 of 18 disagreements) and that were classified as moderate by FPS_SBJ_ but as severe by FPS_SWA_ (5 of 18 disagreements). This suggests that, for juvenile green turtles in Brazil, FPS_SWA_ had a higher agreement with the experts’ subjective assessment of FP manifestation severity than FPS_HWI_.

## Discussion

### Size, number, anatomical distribution and other features of tumors

The total number of tumors varied considerably among specimens (range 1 to 129) and most of them were relatively small, being classified in the size categories A and B (i.e. smaller than 4 cm). Analysis according to size classification of tumors revealed that the average per affected turtle was 8.7±6.8 and 79% of FP-green sea turtles had more than ten tumors. Studies in Indonesia with 4407 green sea turtles (949 with FP tumors) revealed an average of 5±4.1 tumors per individual (range 1 to 29) and only 12.6% had more than ten tumors [22].

The FP tumors exhibited varied morphology, color and texture (smooth to verruciform), as documented in previous studies in Brazil [41]. Most tumors were on the limbs, results consistent with previous studies on green sea turtles in Brazil [21,30,31] and southeastern USA [34]. In Hawaii, tumors are predominantly located in the anterior areas of the body, including the eyes and inside the oral cavity [25–27]. Previous studies found that the histopathological characteristics of tumors may depend on their anatomical positioning [22]. Additionally, unusual histopathological presentations such as renal myxofibromas and cutaneous fibromas have also been documented in some instances [28,42].

### Challenges in quantifying tumors and scoring fibropapillomatosis

In order to study the differences in the clinical manifestations of FP and in the size and distribution of tumors, methods allowing for the quantification and qualification of the disease are necessary. The simplest approach to evaluate the severity of FP is to subjectively classify it as ‘absent’, ‘mild’, ‘moderate’ or ‘severe’ based on the visual inspection of specimens or of dorsal and ventral photographs [4,35,36]. Wood & Wood (1993) [43] proposed the first approach to objectively classify in four scores (grades ‘0’, ‘1’, ‘2’ or ‘3’; with ‘0’ corresponding to the absence of cutaneous FP tumors) based on the maximum size of the tumors and having an additional score level (grade ‘4’) for individuals with tumors that physically hampered movement or caused blindness. More recently, researchers have proposed an alternative classification system that combines the number, size range, location, external morphology and external/internal distribution of tumors to classify green sea turtles into four scores (grades ‘0’, ‘1’, ‘2’ or ‘3’) [34,44].

An alternative approach was developed classifying tumors into four categories depending on their size, and then using the number of tumors in each size category to objectively classify FP [32]. This approach was developed despite some degree of subjectivity and it is considered a reliable index of physiological effects of FP [45,46] and has been broadly used [19,27,31,47]. In parallel, several researchers proceeded to count and describe the anatomical distribution of tumors, especially when they occurred internally [22,25–27]. More recently, studies in Brazil measured each tumor individually and then used these measurements to estimate the total area of tumors for each individual, a method that provided a quantitative index to represent the severity of FP [33]. However, because counting and measuring tumors can be time-consuming in some cases, which may not be compatible with field conditions or animal welfare constraints, simplified methods are necessary to allow for rapid but representative assessment of the size, quantity and distribution of tumors.

In this study, we examined a dataset that combined this four-category size classification proposed by Work and Balazs (1999) [32] with an eleven-category anatomical distribution classification. Obtaining data with this level of detail proved logistically feasible and practical in the field while also providing valuable data for in depth analyses of the clinical manifestations of FP.

The classification system proposed by Work & Balazs (1999) [32] was not meant to be universal, and while it was appropriate for the green sea turtles in Hawaii, problems arise when we attempted to apply it to the same species in the Southwest Atlantic. Our statistical analysis demonstrated that 73% of the studied specimens could not be classified according to Work & Balazs system [32]. When this system was adapted in a manner to allow it to classify all individuals in our dataset, it produced poor agreement with other classification schemes. This is likely the result of the differences in the clinical manifestations of FP between study sites, with green sea turtles in Brazil generally presenting a large number of small tumors whereas in Hawaii they have less numerous but larger tumors.

As a result, in this study we developed and propose a new approach to classify the severity of FP, a fibropapillomatosis index (FPI) based on a weighted sum model that considers the size category and number of tumors. This function can then be used to classify individuals into three ordinal categories of FP severity, the FPS_SWA_, which has substantial agreement with subjective assessments of the severity of FP. The function used to calculate FPI is simple and can be readily calculated in the field, and the FPS_SWA_ classification system is similarly practical. This information is summarized in the S2 File.

Lastly, it is worth noting that FPI and FPS_SWA_ can be readily applied to data from sea turtles studied in other regions of the world. Furthermore, if necessary the function parameters and the score system thresholds can also be easily adjusted to better reflect regional differences in FP manifestation, whilst maintaining a consistent standard of data collection to allow for inter-regional comparisons.

## Conclusions

Our results emphasize the need for studies thoroughly describing and quantifying the number, size and anatomical distribution of FP tumors in sea turtles. Understanding these quantitative and qualitative aspects will be critical to better understand the pathogenesis of FP and the factors that modulate the development of tumors and their impacts on individual health, and to compare the clinical manifestation of FP among different geographic regions. The objective indexing/scoring approaches herein proposed provide a simple yet detailed framework for data collection and analysis of the clinical manifestations of FP, allowing for a classification of FP that tends to be less influenced by observer bias or experience than subjective scores.

## Supporting Information

S1 File Raw data. (XLSX)

S2 File Simplified instructions on how to record the number, size and distribution of FP tumors in sea turtles, and how to obtain FPI and FPS_SWA_. (DOCX)
