# Supplementary material for: Challenges in Evaluating the Severity of Fibropapillomatosis: A Proposal for Objective Index and Score System for Green Sea Turtles (Chelonia mydas) in Brazil
Source: PLoS One. 2016 Dec 9;11(12):e0167632. doi: 10.1371/journal.pone.0167632 (PMC5147950; doi:10.1371/journal.pone.0167632)
Supplement: S2 File — (DOCX) [file pone.0167632.s002.docx]

**Recording the number, size and distribution
of fibropapillomatosis tumors in sea turtles**

1. Classify fibropapillomatosis tumors by size:

| **Category** | **Tumor size** |
| --- | --- |
| A | < 1 cm |
| B | 1 – 4 cm |
| C | > 4 – 10 cm |
| D | > 10 cm |

Work & Balazs 1999 (J. Wildl. Dis. 35:804-807)

1. Count the number of tumors of each size category in each anatomical region:

|  | - Left eye (LE) - Right eye (RE) - Head (HE) - Neck (NE) - Left forelimb (LFL) - Right forelimb (RFL) - Carapace (CA) - Plastron (PL) - Left hindlimb (LHL) - Right hindlimb (RHL) - Inguinal region/tail (IR) |
| --- | --- |

Drawing adapted from Pritchard & Mortimer 1999 (pp. 23-44 in Eckert et al 1999, Research and Management Techniques for the Conservation of Sea Turtles, IUCN/SSC Marine Turtle Specialist Group, Washington, USA)

1. Calculate the sum of tumors that the individual has for each size category (N_A_, N_B_, N_C_ and N_D_), then use them to obtain the individual’s fibropapillomatosis index (FPI):

| **FPI** = 0.1 × **N_A_** + 1 × **N_B_** + 20 × **N_C_** + 40 × **N_D_** |
| --- |

1. Use the value of FPI to obtain the individual’s Southwest Atlantic fibropapillomatosis score (FPS_SWA_):

| **Score** | **FPI** |
| --- | --- |
| mild | < 40 |
| moderate | 40 – 120 |
| severe | ≥ 120 |

**Recording the number, size and distribution
of fibropapillomatosis tumors in sea turtles**

1. Clasifique fibropapilomas por el tamaño:

| **Categoría** | **Tamaño del tumor** |
| --- | --- |
| A | < 1 cm |
| B | 1 – 4 cm |
| C | > 4 – 10 cm |
| D | > 10 cm |

Work & Balazs 1999 (J. Wildl. Dis. 35:804-807)

1. Conte el número de fibropapilomas en cada categoría de tamaño en cada región anatómica:

|  | - Ojo izquierdo (LE) - Ojo derecho (RE) - Cabeza (HE) - Cuello (NE) - Aleta anterior izquierda (LFL) - Aleta anterior derecha (RFL) - Caparazón (CA) - Plastrón (PL) - Aleta poterior izquierda (LHL) - Aleta poterior derecha (RHL) - Región inguinal/cola (IR) |
| --- | --- |

Drawing adapted from Pritchard & Mortimer 1999 (pp. 23-44 in Eckert et al 1999, Research and Management Techniques for the Conservation of Sea Turtles, IUCN/SSC Marine Turtle Specialist Group, Washington, USA)

1. Calculate the sum of tumors that the individual has for each size category (N_A_, N_B_, N_C_ and N_D_), then use them to obtain the individual’s fibropapillomatosis index (FPI):

| **FPI** = 0.1 × **N_A_** + 1 × **N_B_** + 20 × **N_C_** + 40 × **N_D_** |
| --- |

1. Use the value of FPI to obtain the individual’s Southwest Atlantic fibropapillomatosis score (FPS_SWA_):

| **Score** | **FPI** |
| --- | --- |
| mild | < 40 |
| moderate | 40 – 120 |
| severe | ≥ 120 |
